# Understanding breastfeeding behaviours: a cross-sectional analysis of associated factors in Ireland, the United Kingdom and Australia

**DOI:** 10.1186/s13006-020-00344-2

**Published:** 2020-12-02

**Authors:** Danielle Gallegos, Joy Parkinson, Sinead Duane, Christine Domegan, Elena Jansen, Rebekah Russell-Bennett

**Affiliations:** 1 School of Exercise and Nutrition Sciences, Queensland University of Technology, Victoria Park Rd, Kelvin Grove, QLD 4059 Australia; 2 Woolworths Centre for Childhood Nutrition Research, Faculty of Health, Queensland University of Technology, Graham St, South Brisbane, QLD 4101 Australia; 3 Social Marketing @ Griffith, Griffith University, 170 Kessels Rd, Nathan, QLD 4111 Australia; 4 Applied Systems Thinking, Whitaker Institute, National University of Ireland, Galway, Ireland; 5 Division of Child & Adolescent Psychiatry, Department of Psychiatry & Behavioral Sciences, Johns Hopkins University School of Medicine, Baltimore, MD USA; 6 School of Advertising, Marketing and Public Relations, Queensland University of Technology, Gardens Point, Brisbane, 4000 Australia; 7 Centre for Behavioural Economics, Society and Technology, Queensland University of Technology, Gardens Point, Brisbane, 4000 Australia

**Keywords:** Breastfeeding, Policy, Support, Health systems, Ireland, United Kingdom, Australia

## Abstract

**Background:**

Breastfeeding is a complex behaviour relying on a combination of individual mother and infant characteristics, health systems, and family, community and professional support. Optimal breastfeeding in high-income countries is particularly low. Despite having similar sociocultural backgrounds, breastfeeding rates between Ireland, the United Kingdom (UK) and Australia vary, thus there is a need to understand whether this is due to individual, sociocultural or policy differences. This research identifies the between-country differences in infant feeding mode and examines if country differences in feeding mode persist once known individual, behavioural and structural factors are considered using socioecological and person-context models.

**Methods:**

Participants were adult women with at least one infant less than 6 months of age, who completed an online survey (*n* = 2047) that was distributed by social media in June 2016. Within-country differences in infant feeding mode (‘any breastfeeding’ vs. ‘no breastfeeding’) were examined first before hierarchical multivariable logistic regression was used to determine if country differences in feeding mode persisted after adjusting for known factors associated with breastfeeding.

**Results:**

In this sample, ‘any breastfeeding’ rates were 89, 71 and 72% in Australia, Ireland and the United Kingdom respectively. Within-country differences were evident in Australia, Ireland and the UK. Four factors showed no association with infant feeding mode in Australia while they did in the other countries (maternal age, income, skin-to-skin contact, support from friends and family). Two factors were unique to Australia: the odds of being in the ‘no breastfeeding’ group *increased* when the baby was delivered via caesarean and when not enough breastfeeding information was available after birth. One determinant was unique to Ireland: the odds of being in the ‘no breastfeeding’ group *increased* when respondents indicated they were not religious; in the UK this occurred when respondents were living in a town/village. After adjusting for sets of known factors of infant feeding mode based on socioecological and person-context models, country differences remained in hierarchical regressions: the odds of not breastfeeding were higher in both Ireland (AOR 3.3, 95%CI 1.8,6.1) and the United Kingdom (AOR 2.7, 95%CI 1.5, 4.7) compared to Australia.

**Conclusions:**

This study indicates that different levels in the socioecological system are related to infant feeding behaviours. An adequate inter-systems level response would consider the interactions within and between behavioural and structural mechanisms which support breastfeeding behaviour. Optimising infant feeding practices will require an integrated web of interventions that go beyond the individual and focus on addressing factors that will influence families within their communities as they move between systems.

**Supplementary Information:**

The online version contains supplementary material available at 10.1186/s13006-020-00344-2.

## Background

Breastfeeding is a complex behaviour; it relies on individual maternal traits and behaviours as well as infant characteristics intersecting with health systems and services, family and community support, workplace policy and broader cultural values [1]. Breastfeeding has non-modifiable determinants, such as maternal age, socioeconomic status, geographical residence, parity and birthweight of the infant, as well as modifiable factors, such as type of delivery, self-efficacy, attitudes (influenced by religion), previous exposure to breastfeeding and social and professional support [2–6]. An enabling environment for breastfeeding encompasses an understanding of individual mother and infant attributes, and the barriers and facilitators of a range of settings (health systems/services, family, community and workplace), which are all embedded within specific sociocultural and market systems.

Breastfeeding rates (any and exclusive breastfeeding) in high-income countries remain poor [7]. Breastfeeding duration in high-income countries is lower than many low- and middle-income countries, with fewer than one in five children breastfed by the age of 12 months [8]. Assumptions are often made surrounding the homogeneity of behaviour within high-income countries. In Australia, in 2011, initiation (baby put to the breast within an hour of birth) was at 96%, falling to an exclusive breastfeeding rate of 15% and an “any breastfeeding” rate of 60% when infants are 6 months of age [9]. In the Republic of Ireland in 2015, 58% of women were breastfeeding on discharge from hospital at, on average, day two postpartum (a different measure to the baby put to the breast within an hour of birth) and 35% of infants were receiving any breastmilk at 3 months [10]. The 2008 longitudinal national infant feeding survey indicates that 45% of Irish women put the infant to the breast within an hour of birth, but only 2.4% of women were exclusively breastfeeding their infants at 6 months of age [11]. In the United Kingdom (UK) in 2010, breastfeeding was initiated by 81% of mothers, while only 1% were exclusively breastfeeding at 6 months, with 34% undertaking any breastfeeding at the same age [12]. Timely, nationally representative indicators of any and exclusive breastfeeding at 3, 6 and 12 months in these three settings were difficult to obtain, indicating the generally poor monitoring of breastfeeding rates in high-income countries [8].

For breastfeeding duration and exclusivity to improve, a range of strategies have been implemented including appropriate policies related to birthing and the use of formula feeding within the hospital setting, as well as adequate, timely, high-quality antenatal and postnatal counselling from health professionals or peers [13, 14].

Taking a socioecological, systems approach would require the integration of health systems with all the remaining systems, that is, micro-, meso-, exo- and other elements of the macrosystem. The microsystem (micro) includes the settings in which the individual has the most interactions (home, work, school). The mesosystem (meso) concerns the relations between microsystems or connections between contexts (home and health service). The exosystem (exo) links a social setting in which the individual does not have an active role and the individual’s immediate context (parent’s workplace). Finally, the macrosystem (macro) is the overarching institutional patterns of culture in which individuals live, including economic, social, educational, legal and political systems from where micro, meso and exo levels manifest (socioecological model) [15]. Such an approach also identifies both modifiable and non-modifiable system-level factors (that can be categorised as individual, behavioural and structural) that affect individual, community health and policy (person-context model). This approach also requires system and environmental changes to create sustainable organisational and community shifts [16]. A socioecological systems approach posits interventions need integration across systems and settings with social mobilisation and mass media at the meso-level, legislation, policy and monitoring at the macro level, as well as individual counselling, support and management at the individual/micro level [1].

Despite having similar sociocultural backgrounds, health systems and advanced economies, breastfeeding rates between Ireland, the UK and Australia do vary, raising questions as to whether this is due to individual, sociocultural or policy variations. Consequently, this research sought to identify the within-country differences in infant feeding mode in the three high-income industrialised countries and examine if between-country differences existed once known sets of factors associated with breastfeeding were taken into account. In so doing, any system-level variations could pave the way for further research to examine community and policy initiatives that account for these variations.

## Methods

### Design, settings and participants

A cross-sectional study was undertaken. A convenience sample of women with at least one infant less than 6 months of age completed an online self-report survey. The survey consisted of 62 items with skip logic and was administered through Key Survey (v8.7) [17] (Additional file 1). The survey was launched and advertised via a range of parenting-focused Facebook and blog sites in Australia, Ireland and the UK. The survey and the online format was modelled on one previously undertaken in Australia [18]. The survey was open for 2 weeks during June 2016 and participants were actively encouraged to share the link. All prospective participants were provided with information on the length of the survey, where the data would be stored and who had access. Participants were screened using eligibility questions and those not meeting the criteria were thanked and exited from the survey (this data was not captured and is unable to be reported). Eligible mothers were over the age of 18 years, with at least one child under the age of 6 months, with no previous or current diagnoses of mental health issues. Items had a “don’t know”, “do not want to say” option and ethics required that participants were able to skip questions. Submitting the questionnaire online was taken as consent. When exiting the survey, participants were offered a charity donation to one of three relevant charities for participation. On closure of the survey all IP addresses were checked to ensure that only unique surveys were included, there were no duplicates. The Checklist for Reporting Results of Internet E-Surveys (CHERRIES) was used to report this information (see Additional file 2) [19].

### Variables

Participants reported on a range of sociodemographic characteristics known to impact on breastfeeding variables as well as feeding-specific variables. These were categorised as micro-, meso-, exo- and macro-system factors (i.e. the socioecological model) as well as individual, behavioural or structural factors (i.e. the person-context model) as outlined in Table 1.

**Table 1:** Categorisation of variables

Socioecological level	Definition	Construct		
		Individual	Behavioural	Structural
Microsystem /Individual	Biological and personal history factors	Maternal age Parity Mother education level Income Type of birth	Fed yourself as a baby First food attitudes	Feeding knowledge
Mesosystem	Relationships/ interpersonal factors		Support level	Encouraged by professional
Exosystem	Community and organizational factors		Feeding intention	Religiousness Skin-to-skin contact Urban living Feeding information
Macrosystem	Social policy, culture, societal attitudes and beliefs			Country/ region

#### Sociodemographic characteristics

Sociodemographic data included maternal age, ethnicity, education level, income, urbanisation of home town, and religiousness (categorised as belonging to a church, denomination or religious community ‘yes’ vs. ‘no’). Questions were drawn from the country census data collection and then pooled to enable cross-country categorisation. Ethnicity was collected as per the census in each country, for example in Ireland, included Irish, Irish traveller, any other Irish background etc. In Australia questions related to indigeneity and then cultural background for non-Indigenous were included. Comparisons were then made with “white ethnicity” across all countries and with those born outside of each country. Gross household income was broken down into quintiles for each country, higher values indicating higher incomes, these were then categorised into eight segments to represent respective income brackets with “low income” representing the lowest to fourth segments across countries. Additionally, respondents indicated whether the study child was their first child (categorised as first child ‘yes’ vs. ‘no’) and the delivery of this child (categorised as ‘vaginal’ vs. ‘elective/planned or emergency caesarean’). Additional details about the baby’s birth were asked, including birth weight, place of birth, skin-to-skin contact after birth (initiation) and timing of first feed.

#### Feeding-specific variables

Participants were also asked how they were fed as a baby, feeding intentions and how long they planned to breastfeed. Infant feeding was further investigated by gauging current (at time of survey) feeding of their baby and plans to feed their baby over the next 4 weeks.

To measure constructs, empirically validated scales were used and adapted to suit the country context (for example terms used to describe place of birth); where scales did not exist, items were created for the purpose of the study. *Feeding knowledge* related to duration and exclusivity was assessed by asking respondents at which age infants should be introduced to other foods or solids (categorised as ‘correct’ = around 6 months vs. ‘incorrect’ = all other responses). This knowledge question directly relates to the predominant public health messaging used in each country. *Feeding attitude* was assessed with the Iowa Infant Feeding Attitudes Scale [20]. The mean score was calculated for the 17 items (7-point Likert scale, with 1 = strongly disagree to 7 = strongly agree) if at least six items were completed. One item was omitted from the original scale (‘Mothers who formula-feed miss one of the great joys of motherhood’), while another item was added (‘Bottle feeding increases father–infant bonding’). This change, while not validated, was made to reduce the potential criticism related to judgemental constructs this statement has evoked previously in bottle-feeders. Despite the change, the alternate questions’ internal consistency was acceptable (*a* = .81). Approximately half of the items were negatively worded, and therefore were reversed before the scale was summated. Total scores ranged from 17 (positive attitudes towards formula feeding) to 85 (positive attitudes to breastfeeding). A score of 51 was neutral.

*Feeding support* determined the level of social support received from a range of sources on a 7-point Likert scale (0 = no support, 6 = lots of support). Items were arranged into three groups and averaged with higher scores indicating more support. The first group included support from family and friends (i.e. baby’s father, own mother, other relatives, best friend, other friends; *a* = 0.86). The second group included support from health professionals (i.e. doctor, midwife or health visitor, other health professionals, classes, support groups; *a* = 0.93). The third group included support through technology (i.e. websites, mobile phone apps, written or video material; *a* = 0.92).

Respondents were asked three questions to assess *breastfeeding encouragement* after birth. Firstly, ‘What kind of food did the baby receive for the first feed after birth?’ (categorised as ‘breastmilk’ vs. ‘formula’). Secondly, ‘After the birth was the baby given opportunities to independently find the breast by being placed in skin-to-skin contact with the mother where the baby could move freely?’ (categorised as ‘yes’ vs. ‘no’). Thirdly, ‘How soon after your baby was born were you encouraged by a health care professional (doctor, nurse, midwife etc.) to breastfeed your child?’ (categorised as ‘within 30mins’ [immediately or within a few minutes plus > few minutes to 30mins] vs. ‘longer than 30mins or not encouraged’ [>30mins to 1 h plus > 1 h to 2 h plus > 2 h to 24 h plus not encouraged plus don’t know/can’t say]).

*Feeding history, plan and information* formed the last set of variables. Respondents were asked how they were fed as a baby (categorised as ‘just breastmilk and nothing else for at least 4–6 months’ vs. ‘other’ [mainly breastmilk with some formula, mainly fed with formula with breastmilk only sometimes, just formula, don’t know]) and how they had planned to feed their baby before the baby was born (categorised as ‘breastfeed’ vs. ‘other’ [formula feed, combination of breast and formula feeding, I hadn’t decided]). They were also asked whether they received enough information about a) breastfeeding and b) formula feeding after their baby was born (categorised as ‘yes’ vs. ‘no’).

#### Current feeding mode (dependent variable)

Questions related to breastfeeding initiation, duration and exclusivity were drawn from the Australian Infant Feeding Survey [9]. Participants’ current feeding mode was categorised into ‘any breastfeeding’ = exclusive, predominant, complementary or any breastfeeding (including mixed feeding with formula) vs. ‘no breastfeeding’ = formula feeding only.

### Data analysis

Analyses were conducted in IBM SPSS Statistics version 23. Descriptive statistics were examined and differences between the three countries tested using an analysis of variance (ANOVA) for continuous variables (Likert scales) and the Chi-Square test for categorical variables. Next, bivariate logistic regression analyses were performed to identify factors associated with infant feeding mode (i.e. dependent variable, 0 = any breastfeeding [reference group], 1 = no breastfeeding) by country (i.e. within-country differences) as well as for the overall sample. It should be noted that while we use “country” the UK is made up of four individual countries, England, Northern Ireland, Scotland and Wales but for the purposes of analysis were combined into the UK. Significant variables (based on 95% CI) from the overall sample were selected and then combined into two hierarchical multivariable logistic regression models to evaluate the change in variance explained. For the first model, reflecting the socioecological classification, variables were entered in the following order: Step 1 – microsystem, Step 2 – mesosystem, Step 3 – exosystem, and Step 4 – macrosystem. For the second model, reflecting the person-context classification, variables were entered as follows: Step 1 – individual, Step 2 – behavioural, Step 3 – structural, and Step 4 – countries. Notably, country of residence was entered in the final step for both models to specifically determine if differences in feeding mode existed across the different countries, after adjusting for all other sets of variables. Missing data were deleted listwise for regression analyses.

## Results

A total of 2068 participants completed or partially completed the survey. All mothers living outside the designated countries (Ireland, Australia, the UK) and those who did not complete the survey (did not press submit and therefore consent not given) were removed (*n* = 18). An additional three mothers who were only feeding their infants complementary foods were also removed, leaving a total of 2047 respondents.

The sample comprised women from the UK (40.9%), with Australian (31.3%) and Irish (27.8%) women representing a smaller but approximately equal portion. On average, women were 31 years old (Australia = 30, Ireland = 33, UK = 31) and all women stated they had infants under the age of 6 months. Due to an administrative error infant’s birth date was omitted and no further information on baby age is available. The majority of respondents were born in Europe (*n* = 1387, 68.4%) and Australia (*n* = 576, 28.4%). Smaller numbers of women had been born in Asia (*n* = 23, 1.1%), Africa (*n* = 16, 0.8%), North America (*n* = 16, 0.8%) and South America (*n* = 9, 0.4%). Public hospitals were the main place of delivery (*n* = 1753, 85.8%), with 10.7% of births taking place in private hospitals (*n* = 219) and 2.5% at home (*n* = 51). Private hospitals were more commonly cited as a place of delivery in Australia (30.1%) compared to the other countries (4.3–4.6%), where almost all births occurred in public hospitals (> 92%).

Sample characteristics reported by country of residence are presented in Table 2. Differences across the three countries were evident for the majority of variables.

**Table 2:** Descriptive statistics and differences between the three countries

		Australia (*n* = 641)^a^ N (%) or M ± SD	Ireland (*n* = 568)^a^ N (%) or M ± SD	UK (*n* = 838)^a^ N (%) or M ± SD	χ^2^ (df), effect size (*Phi or Cramer’s V)*	*p*-value
Dependent variable						
Infant feeding mode	Any BF No BF	568 (88.6) 73 (11.4)	402 (70.8) 166 (29.2)	604 (72.1) 234 (27.9)	72.5 (2), 0.19	< 0.001
Independent variables—demographic characteristics						
Maternal age		30 ± 4^a^	33 ± 4^a,b^	30 ± 4^b^		< 0.001
Parity	First child Second+ child	366 (57.2) 274 (42.8)	252 (44.4) 315 (55.6)	483 (57.6) 355 (42.4)	27.9 (2), 0.12	< 0.001
Education level	University No university	406 (63.3) 235 (36.7)	409 (73.2) 150 (26.8)	550 (66.6) 276 (33.4)	13.5 (2), 0.08	< 0.001
Income (quintiles)	Lowest to 4th 4th to highest	283 (46.0) 332 (54.0)	198 (37.2) 334 (62.8)	457 (57.3) 340 (42.7)	53.5 (2), 0.17	< 0.001
Religiousness	Yes No	230 (39.2) 357 (60.8)	409 (77.0) 122 (23.0)	298 (37.3) 501 (62.7)	233.3 (2), 0.35	< 0.001
Urban living	Yes (city) No (other)	335 (52.8) 300 (47.2)	162 (28.8) 401 (71.2)	212 (25.3) 625 (74.7)	132.3 (2), 0.26	< 0.001
Type of birth	Vaginal Planned C Emergency C	445 (69.9) 100 (15.7) 92 (14.4)	395 (70.3) 86 (15.3) 81 (14.4)	605 (72.6) 85 (10.2) 143 (17.2)	13.3 (4), 0.08	0.01
Independent variables—feeding-specific variables						
Feeding knowledge	Correct Incorrect	355 (56.7) 271 (43.3)	362 (65.9) 187 (34.1)	583 (71.4) 233 (28.6)	34.1 (2), 0.13	< 0.001
Feeding attitudes		5.41 ± 0.82^a^	5.37 ± 0.95	5.28 ± 0.97^a^		0.023
Support level	Group 1 (F&F)	22.36 ± 7.82^a, b^	19.77 ± 8.32^a^	19.67 ± 8.28^b^		< 0.001
	Group 2 (Prof)	17.78 ± 8.98^a, b^	14.75 ± 9.17^a, c^	11.81 ± 8.66^b, c^		< 0.001
	Group 3 (Tech)	10.17 ± 6.07^a^	9.44 ± 5.73^b^	8.13 ± 5.81^a, b^		< 0.001
Fed yourself as a baby	Just breastmilk	361 (56.6)	149 (26.2)	333 (39.9)	182.3 (8), 0.30	< 0.001
	Mainly BM	58 (9.1)	72 (12.7)	128 (15.3)		
	Mainly formula	69 (10.8)	43 (7.6)	80 (9.6)		
	Just formula	116 (18.2)	286 (50.4)	266 (31.9)		
	Don’t know	34 (5.3)	18 (3.2)	28 (3.4)		
First food	Breastmilk	594 (93.2)	484 (85.5)	741 (88.8)	19.1 (2), 0.097	< 0.001,
	Formula	43 (6.8)	82 (14.5)	93 (11.2)		
Skin-to-skin contact to find breast	Yes	484 (75.9)	401 (71.6)	606 (72.4)	9.0 (4), 0.07	0.061
	No	139 (21.8)	129 (23.0)	201 (24.0)		
	Don’t know	15 (2.4)	30 (5.4)	30 (3.6)		
Encouraged by professional	Immediately/ within few mins	281 (44.7)	243 (43.2)	268 (32.2)	82.1 (12), 0.20	< 0.001
	>few mins to 30mins	145 (23.1)	101 (17.9)	193 (23.2)		
	>30mins to 1 h	84 (13.4)	47 (8.3)	104 (12.5)		
	> 1 h to 2 h	43 (6.8)	34 (6.0)	60 (7.2)		
	> 2 h to 24 h	40 (6.4)	35 (6.2)	56 (6.7)		
	Not encouraged	22 (3.5)	81 (14.4)	124 (14.9)		
	Don’t know	14 (2.2)	22 (3.9)	27 (3.2)		
Feeding plan	BF	590 (92.2)	451 (79.7)	675 (80.5)	58.5 (6), 0.17	< 0.001
	FF	18 (2.8)	58 (10.2)	61 (7.3)		
	Combo	19 (3.0)	29 (5.1)	69 (8.2)		
	Not decided	13 (2.0)	28 (4.9)	33 (3.9)		
Feeding information	Breastfeeding Yes No	568 (89.4) 67 (10.6)	463 (82.1) 101 (17.9)	677 (81.3) 156 (18.7)	20.2 (2), 0.10	< 0.001
	Formula feeding Yes No	342 (56.6) 262 (43.4)	403 (76.9) 121 (23.1)	478 (60.1) 318 (39.9)	57.1 (2), 0.17	< 0.001

*BF* Breastfed, *FF* Formula fed, *Combo* Combination of BF and FF, *Planned C* Planned caesarean, *Emergency C* Emergency caesarean, *Pred/Exclu* Predominantly/exclusively

Note: results exclude ‘don’t know’ responses; superscripts indicate groups that differed from one another

^a^Sample sizes vary for each item due to missing data

Table 3 provides an overview of the univariate relationships between potential factors and feeding mode. For the overall sample the majority of variables were associated with feeding mode. There were three exceptions. Parity, religiousness and ‘enough information about formula feeding after birth’ had odds ratios with confidence intervals including one. These variables were consequently not carried forward into the hierarchical multivariable regression analyses. Looking at the country-specific univariate relationships, it appeared that relationships for six potential factors varied for Australia, compared to Ireland or the UK. Four factors showed no association with feeding mode in Australia while they did in the other countries (i.e. maternal age, income, skin-to-skin contact, support from friends and family). Two factors were unique to Australia: the odds of being in the ‘no breastfeeding’ group *increased* when the baby was delivered via caesarean (OR 2.4, 95%CI 1.4, 3.9) and *decreased* when enough breastfeeding information was available after birth (OR 2.1, 95%CI 1.1, 4.1). One determinant was unique to Ireland: the odds of being in the ‘no breastfeeding’ group *increased* when respondents indicated their non-religiousness (i.e. did not belong to a church, denomination or religious community; OR 2.9, 95%CI 1.9, 4.3). Similarly, one determinant was unique to the UK: the odds of being in the ‘no breastfeeding’ group *increased* when respondents were living in a town or village (OR 2.1, 95%CI 1.4, 3.1).

**Table 3:** Univariate relationships between infant feeding mode (0 = any breastfeeding vs. 1 = no breastfeeding) and potential factors by country and for the overall sample (*N* = 2047)

Any breastfeeding (reference group)	Australia (*n* = 641) OR (95%CI)	Ireland (*n* = 568) OR (95%CI)	UK (*n* = 838) OR (95%CI)	Total sample OR (95%CI)
Maternal age	0.96 (0.90, 1.02)	**0.94 (0.90, 0.99)**	**0.91 (0.87, 0.95)**	**0.95 (0.92, 0.97)**
First child (no)^a^	1.23 (0.75, 2.03)	1.12 (0.78, 1.61)	1.31 (0.97, 1.79)	1.16 (0.94, 1.427)
Income (fourth to highest quintile)	1.50 (0.91, 2.46)	**1.75 (1.19, 2.56)**	**2.28 (1.64, 3.176)**	**1.88 (1.52, 2.33)**
Education level (university)	**2.47 (1.52, 4.05)**	**2.85 (1.92, 4.23)**	**2.604 (1.90, 3.57)**	**2.36 (1.91, 2.92)**
Religiousness (yes)	1.17 (0.68, 2.00)	**2.86 (1.876, 4.348)**	1.099 (0.80, 1.52)	1.19 (0.96, 1.47)
Urbanisation (city)	1.03 (0.63, 1.68)	1.37 (0.90, 2.07)	**2.076 (1.41, 3.07)**	**1.86 (1.47, 2.35)**
Delivery (vaginal)	**2.37 (1.44, 3.91)**	1.305 (0.88, 1.93)	1.237 (0.89, 1.73)	**1.39 (1.11, 1.73)**
Feeding intention (breastfeed)	**11.55 (6.16, 21.68)**	**14.63 (8.96, 23.89)**	**7.947 (5.46, 11.56)**	**11.41 (8.75, 14.87)**
Feeding knowledge (correct)	**2.36 (1.417, 3.91)**	**2.56 (1.75, 3.76)**	**2.086 (1.51, 2.89)**	**1.94 (1.57, 2.40)**
Feeding attitudes mean	**0.12 (0.08, 0.19)**	**0.12 (0.09, 0.18)**	**0.126 (0.09, 0.17)**	**0.13 (0.12, 0.16)**
First food after birth (breastmilk)	**15.05 (7.68, 29.49)**	**26.78 (13.65, 52.53)**	**14.487 (8.49, 24.71)**	**17.68 (12.56, 24.87)**
Skin to skin contact after birth (yes)	1.60 (0.94, 2.72)	**2.17 (1.47, 3.20)**	**1.69 (1.22, 2.34)**	**1.85 (1.48, 2.31)**
Breastfeeding encouragement by professional (≤30mins)	**2.26 (1.37, 3.72)**	**2.74 (1.89, 3.98)**	**2.14 (1.58, 2.92)**	**2.47 (2.00, 3.05)**
Mum fed as baby (breastmilk)	**3.24 (1.92, 5.46)**	**3.23 (1.95, 5.34)**	**2.515 (1.80, 3.53)**	**3.23 (2.54, 4.11)**
Had enough info after birth about breastfeeding (yes)	**2.12 (1.09, 4.11)**	1.53 (0.97, 2.41)	0.98 (0.67, 1.45)	**1.44 (1.10, 1.88)**
Had enough info after birth about formula feeding (yes)	0.63 (0.376, 1.065)	1.17 (0.76, 1.80)	1.02 (0.75, 1.39)	0.86 (0.69, 1.08)
Support—friends & family	0.98 (0.95, 1.01)	**0.95 (0.93, 0.98)**	0.98 (0.96, 1.00)	**0.97 (0.95, 0.98)**
Support—health professionals & groups	**0.93 (0.90, 0.96)**	**0.94 (0.92, 0.96)**	**0.94 (0.92, 0.96)**	**0.93 (0.92, 0.94)**
Support—technology	**0.88 (0.83, 0.93)**	**0.88 (0.85, 0.92)**	**0.92 (0.89, 0.95)**	**0.90 (0.88, 0.92)**
Country (Australia vs. Ireland)	NA	NA	NA	**3.21 (2.37, 4.35)**
Country (Australia vs. UK)	NA	NA	NA	**3.01 (2.26, 4.02)**

Significant results based on 95% CI are presented in bold

^a^Reference groups (coded as 0) are presented in brackets

Tables 4 and 5 show the results of the hierarchical multivariable logistic regression analyses. For the socioecological model, adding each respective system level added significantly to the variance explained in feeding mode. Significant factors associated with feeding mode were mostly present in the micro-, meso-, and macrosystem levels. Similarly, for the person-context model, adding each level of variables added significantly to the variance explained in feeding mode. Factors mostly from the behavioural level were significantly associated with feeding mode. The overall models explained 59.9% of the variance in feeding mode. After adjustment of all other factors, country differences existed in the odds for mothers to be in the ‘no breastfeeding’, at risk feeding mode group. The odds for being in the ‘no breastfeeding’ group were higher in both Ireland (OR 3.3, 95%CI 1.8, 6.1) and the UK (OR 2.7, 95%CI 1.5, 4.7), compared to Australia.

**Table 4:** Hierarchical multivariable logistic regression for infant feeding mode (0 = any breastfeeding vs. 1 = no breastfeeding) and four levels of factors according to the socioecological model

Variables^a, b^	B	W*χ*^2^	AOR	95% CI
**Step 1 – Microsystem**	Nagelkerke *R*^2^ = 52.6%			
Age	−0.03	1.37	0.97	0.91, 1.02
Income (fourth to highest quintile)	0.10	0.20	1.11	0.70, 1.75
Education level (university)	0.72	9.35*	2.06	1.30, 3.27
Feeding knowledge (correct)	0.30	1.96	1.35	0.89, 2.06
Delivery (vaginal)	0.29	1.55	1.33	0.85, 2.10
Mum fed as baby (breastmilk)	0.81	12.98*	2.26	1.45, 3.51
First food after birth (breastmilk)	1.52	18.75*	4.55	2.29, 9.04
Feeding attitudes mean	−1.80	152.87*	0.17	0.13, 0.22
**Step 2 – Mesosystem**	Nagelkerke *R*^2^ = 56.2%			
Support—friends & family	0.001	0.003	1.00	0.97, 1.03
Support—health professionals & groups	−0.007	0.20	0.99	0.96, 1.02
Support—technology	− 0.091	14.65*	0.91	0.87, 0.96
Breastfeeding encouragement by professional (≤ 30mins)	−0.03	0.02	0.97	0.62, 1.53
**Step 3 – Exosystem**	Nagelkerke *R*^2^ = 58.6%			
Urbanisation (city)	0.69	8.40*	1.99	1.25, 3.18
Skin to skin contact after birth (yes)	−0.23	0.90	0.79	0.49, 1.28
Feeding intention (breastfeed)	0.75	7.77*	2.12	1.25, 3.59
Had enough info after birth about breastfeeding (yes)	0.45	3.29*	1.57	0.96, 2.57
**Step 4 – Macrosystem**	Nagelkerke *R*^2^ = 59.9% Goodness of fit *χ*^2^ (df) = 634.84 (18)*			
Country (0 = Australia vs. 1 = Ireland)	1.21	15.32*	3.35	1.83, 6.13
Country (0 = Australia vs. 1 = UK)	0.98	11.45*	2.68	1.51, 4.73

*Abbreviations*: *B* unstandardised regression coefficient, *Wχ*^2^ Wald *χ*^2^-test, *AOR* Adjusted Odds ratio, *95% CI* 95% CI for adjusted Odds ratio

*p < 0.05

^a^ Values presented are taken from final model

^b^Reference groups (coded as 0) are presented in brackets

**Table 5:** Hierarchical multivariable logistic regression for infant feeding mode (0 = any breastfeeding vs. 1 = no breastfeeding) and four levels of factors according to the person-context model

Variables^a, b^	B	W*χ*^2^	AOR	95% CI
**Step 1 – Individual**	Nagelkerke *R*^2^ = 5.8%			
Age	− 0.03	1.37	0.97	0.91, 1.02
Income (fourth to highest quintile)	0.10	0.20	1.11	0.70, 1.75
Education level (university)	0.72	9.35*	2.06	1.30, 3.27
**Step 2 – Behavioural**	Nagelkerke *R*^2^ = 56.7%			
Delivery (vaginal)	0.29	1.55	1.33	0.85, 2.10
Mum fed as baby (breastmilk)	0.81	12.98*	2.26	1.45, 3.51
First food after birth (breastmilk)	1.52	18.75*	4.55	2.29, 9.04
Feeding attitudes mean	−1.80	152.87*	0.17	0.13, 0.22
Feeding intention (breastfeed)	0.75	7.77*	2.12	1.25, 3.59
Support—friends & family	0.001	0.003	1.00	0.97, 1.03
Support—health professionals & groups	−0.007	0.20	0.99	0.96, 1.02
Support—technology	−0.09	14.647*	0.91	0.87, 0.96
**Step 3 – Structural**	Nagelkerke *R*^2^ = 58.6%			
Urbanisation (city)	0.69	8.40*	1.99	1.25, 3.18
Skin to skin contact after birth (yes)	−0.23	0.90	0.79	0.49, 1.28
Had enough info after birth about breastfeeding (yes)	0.45	3.29*	1.57	0.96, 2.57
Feeding knowledge (correct)	0.30	1.96	1.35	0.89, 2.06
Breastfeeding encouragement by professional (≤ 30mins)	−0.03	0.02	0.97	0.62, 1.53
**Step 4 – Country**	Nagelkerke *R*^2^ = 59.9% Goodness of fit *χ*^2^ (df) = 634.84 (18)*			
Country (0 = Australia vs. 1 = Ireland)	1.21	15.32*	3.35	1.83, 6.13
Country (0 = Australia vs. 1 = UK)	0.98	11.45*	2.68	1.51, 4.73

*Abbreviations*: *B* unstandardised regression coefficient, *Wχ*^2^ Wald *χ*^2^-test, *AOR* Adjusted Odds ratio, *95% CI* 95% CI for adjusted Odds ratio

* *p* < 0.05

^a^Values presented are taken from final model

^b^Reference groups (coded as 0) are presented in brackets

## Discussion

The data indicate that there are similarities and differences related to breastfeeding behaviours across the three countries. The odds for being in the ‘no breastfeeding’ group were higher in both Ireland and the UK, compared to Australia.

To identify the factors associated with breastfeeding behaviour we examined the data using two approaches, one examining the socioecological systems and the other examining the person-context model. Findings were similar using both approaches. Our analysis using the socioecological systems approach indicates that after taking into consideration micro, meso, and exo factors, country-level differences persist inferring that there are context-specific factors that need to be considered when designing interventions. For example, having enough breastfeeding information after birth and whether a mother lives in an urban area or not. This may indicate that mothers living in non-urban areas require different types of information and support to encourage breastfeeding and there may be other factors such as culture influencing breastfeeding behaviour. This further supports the need for consideration of context when developing breastfeeding interventions. Likewise, when considering factors according to the person-context model that is, whether these are individual, behavioural or structural, factors mostly from the behavioural level were associated with breastfeeding. Only one individual and two structural factors remained significant, while four behavioural factors remained significant. This indicates that behavioural level factors including how the mother was fed as a baby herself, feeding breastmilk as the first food after birth and receiving support via technology are potentially relevant behaviours. There is recognition of many contributing factors to breastfeeding including: an array of systemic factors, supported in some countries with legislation (for example, paid maternity leave, anti-discrimination around lactation breaks and breastfeeding in public spaces); training of health professionals; and introduction of health-service system changes, for example the Baby Friendly Hospital Initiative [1, 21]. However, a majority of breastfeeding interventions tend to focus on individual maternal (intention, self-efficacy and knowledge) and micro-level (family, health professional support) factors in an attempt to rectify sub-optimal infant feeding practices [1]. The findings from this research indicate that factors within and across each socioecological system may need addressing, and that these systems appear to be interdependent and dynamic.

Upon examination of country-specific univariate relationships, it appeared that relationships for six potential factors varied for Australia, compared to Ireland or the UK. In Australia, maternal age, income, skin-to-skin contact and support from friends and family were not associated with infant feeding practice. This result contradicts previous studies regarding age and income and may be a result of low numbers of women in the sample from younger and lower income groups [22, 23]. Skin-to-skin contact is almost ubiquitous in Australia with rates ranging from 72 to 94% cited, the non-association with infant feeding practice indicates that skin-to-skin contact policy alone may not be enough to influence ongoing breastfeeding practice [24]. The two factors unique to Australia: the odds of being in the ‘no breastfeeding’ group *increased* when the baby was delivered via caesarean and *decreased* when enough breastfeeding information was available after birth were as expected. The odds of ‘any breastfeeding’ decrease in infants delivered by caesarean, however there is no difference at 6 months in feeding mode (‘any breastfeeding’ vs ‘no breastfeeding’) if initiation has been undertaken [25]. In addition, women who are more educated are more likely to have a caesarean delivery and therefore may also be more likely to seek out individualised assistance postnatally. Women in Australia who had enough information on breastfeeding after birth were more likely to undergo ‘any breastfeeding’. This finding may lie in the subjective determination of “enough” information, which may vary cross-culturally. While support from health professionals did not remain statistically significant (95%CI) in both models, it is worthy to note that all forms of support from family, friends and health professionals were lower in the UK and Ireland. Support via technology remained significant in both models, which may indicate this is an effective way to provide breastfeeding support to mothers both before and after birth.

One determinant was unique to Ireland: the odds of being in the ‘any breastfeeding’ group *increased* when respondents indicated they were religious (i.e. belong to a church, denomination or religious community). This is contrary to previous work where religion, specifically Catholicism, has been shown to negatively influence breastfeeding initiation rates [6]. This result may, in part, be due to the question asked; religiosity in some countries may have little to do with “belonging” to a church. Religiousness was not included in the final model and so this finding needs to be interpreted with caution due to other potential confounding factors. As other studies have recommended, interventions influencing this exosystem level must reflect the sociocultural norms that manifest in Irish society [6]. The odds of being in the ‘no breastfeeding’ group *increased* when respondents were living in towns or villages in the UK. This may be due to a number of reasons including perceptions of satisfaction with the neighbourhoods in which women live (women less satisfied are less likely to breastfeed) [26] and significant variability of breastfeeding support across the UK [27]. Feeding attitudes tended towards the positive with differences between Australia and the UK, and this variable was statistically significant (95%CI) in the model; women with more positive attitudes were more likely to breastfeed.

This study explored health service factors. However, factors related to returning to work while breastfeeding or breastfeeding in public, which could also be indicative of systemic societal barriers, were not explored. At the individual and behavioural level, if the baby’s first food was breastmilk, the odds of being in the ‘no breastfeeding’ group decreased in all three countries; this may be more important than skin-to-skin contact. This remained statistically significant (95%CI) between countries as a factor associated with breastfeeding behaviour.

This research indicates that after taking individual and structural factors into consideration breastfeeding rates still vary between countries and the odds of being in the ‘no breastfeeding’ group are higher in Ireland and the UK. This points to the potential for inadequate inter-systems-level responses to ensure optimal infant feeding. An adequate inter-systems level response would consider the dynamic interactions within and between behavioural and structural mechanisms which support breastfeeding behaviour. This may include encouraging breastmilk as the first food, providing technology-assisted support before and after birth, and consistent breastfeeding information after birth in a variety of mediums. This is consistent with the large body of research identifying the importance of skin-to-skin contact, breastmilk as the first food, and antenatal and postnatal information and support [2, 28, 29]. In Ireland and the UK in particular, integrated models of information provision and support that are accessible to women at the right time and right place will be an important strategy to improve breastfeeding rates.

Without strategies that normalise breastfeeding as a cultural and social norm, breastfeeding in all three countries is likely to continue to be sub-optimal. All three countries have had attempts at broad breastfeeding strategies; for example, the Australian National Breastfeeding Strategy [30], Breastfeeding in a Healthy Ireland [31], Breastfeeding—A Great Start: A Strategy for Northern Ireland [32] and the introduction of the Baby-Friendly Hospital Initiative [33]. These strategies have been hampered by inadequate implementation frameworks and funding, instead being reliant on individuals and organisations to sustain practice. There has also been a siloed approach to systems change, with separate strategies focused on healthcare, workplaces and education with little cross-system integration. Little attention has been placed on changing social and cultural norms. Consequently, in addition to legislative approaches (paid maternity leave, anti-discrimination), social marketing campaigns that aim to change attitudes towards breastfeeding to engender a normative breastfeeding culture are urgently required that go beyond mass media campaigns. Accessible personalised information and support, which includes that which is delivered via technology—antenatally and postnatally—to enable women to proactively problem-solve will also be essential [18, 34–36].

This study has a number of limitations. The sample was self-selected and given the high level of breastfeeding was skewed to be pro-breastfeeding and well-educated mothers; there was a reasonable spread over income brackets, but women were more likely to have higher rather than lower incomes. There are other variables potentially associated with breastfeeding that have not been taken into consideration. The survey was Facebook-generated, limiting access to women without the internet or social media accounts. This was a cross-sectional, self-reported study, and duration and exclusivity of breastfeeding was not able to be assessed. An examination of back to work policies and procedures was beyond the scope of this study. The sample, however, was large and drew from the community across three countries. Due to the nature of the included variables there may be an issue with multicollinearity. This survey was one-dimensional, only examining the issue from the mothers’ perspective, it did not examine perspectives from other members of the system.

## Conclusions

Women in Ireland and the UK are less likely to breastfeed when compared to women in Australia. Context is therefore important, indicating breastfeeding interventions need tailoring for given contexts, reflecting on local cultural and societal norms which might be impacting on behaviours. These interventions also need to be responsive to take into consideration the interaction between systems. The key contribution of this research is that it builds on previous studies and clearly evidences that the factors associated with breastfeeding are multi-faceted and complex, and vary between countries - with implications that any successful interventions and strategies to improve breastfeeding rates must be tailored to local needs as well as holistically approach the different systems around the mother and infant. Work needs to continue on building individual self-efficacy and knowledge; however, changing breastfeeding behaviour will require an integrated web of multiple strategies that go beyond the individual to address underlying factors that will support families within their communities to optimise infant feeding practices and ultimately child outcomes.

## Supplementary Information

**Additional file 1.** Irish Infant Feeding Survey.

**Additional file 2.** Checklist for Reporting Results of Internet E-Surveys (CHERRIES).

**Additional file 3.** Example Facebook advertisement.

## Data Availability

The datasets used and/or analysed during the current study are available from the corresponding author on reasonable request.
