# Supplementary material for: Understanding breastfeeding behaviours: a cross-sectional analysis of associated factors in Ireland, the United Kingdom and Australia
Source: Int Breastfeed J. 2020 Dec 2;15:103. doi: 10.1186/s13006-020-00344-2 (PMC7709394; doi:10.1186/s13006-020-00344-2)
Supplement: Supplementary file 1 — Additional file 1. Irish Infant Feeding Survey. [file 13006_2020_344_MOESM1_ESM.pdf]

Queensland University of Technology
Brisbane Australia

NUI Galway
OÉ Gaillimh

IRISH INFANT FEEDING SURVEY (OTHER VERSIONS AVAILABLE ON REQUEST)

2015

1. About you

1.1 What is your date of birth? DD/MM/YYYY

1.2 In which country were you born?

1.3 What is your nationality?[1]

1.4 What is your ethnic or cultural background?[1]

1.5 In what country do you live most of the time?

1.6 How would describe where you live?[1]
City
Town
VillageRural
Island
Other <please state>

1.7 What is your current relationship status?[1]
Single, never married
Single, but cohabiting with a significant other
Married
In a domestic partnership or civil union
Divorced
Widowed
Separated

1.8 What is the main language spoken at home?[1]

1.9 Have you ever belonged to any church, denomination, or a religious community? [1]
Yes
No [SKIP TO Q1.11]

1.10 Which one?
Protestant
Roman Catholic
Eastern Orthodox
Islamic
Jewish
Buddhist
Hindu
Other (Write in) ....………………………
(Do not know) (No answer)

1.11 What is your highest level of education training?[1]

1.12 Approximately, what was your total gross household income (before tax) for the past 12 months?[2]
Less than 25, 000
25, 000 001 - 50, 000
50, 001 - 75, 000
Over 75, 001 - 100,000

More than 100,000
Don't know
Don't want to say

1.13 Which best describes your employment BEFORE having this baby
[Drop down box]
Looking after family/home
Student
Part-time employee
Full-time employee
Contract employee
Self-employed
Unemployed
Other <please specify>

1.14 Are you currently on maternity leave? YES/NO
If YES: How long are you taking? <Insert time in months>

1.15 What medical benefits are you currently receiving? **IRELAND ONLY**

2 About your baby's birth

For the rest of this survey we will be asking you questions about your YOUNGEST child who was born within the LAST 6 MONTHS.

Questions asking about breastmilk includes colostrum, expressed breastmilk and breastmilk from a donor or milk bank. When we ask about infant formula this includes infant formula, follow-on formula, soy formula and lactose-free formula.

Please answer all questions as truthfully as possible.

2.1 Is this your first child?
Yes [skip to 2.3]
No

2.2 How many children, including this baby, do you have? <insert number>

2.3 How did you feed your previous children up until 6 months?

	Breast Only	Formula Only	Combination of breast and formula
Eldest Child			
Second Eldest Child			
Third Eldest Child			
Fourth eldest			

2.4 A Is your youngest baby one of a multiple birth?
YES
NO Skip to 2.5

2.4 B If YES, Did you have
[Drop down box]
Twins
Triplets
Other <please state>

IF YOUR LAST PREGNANCY WAS A MULTIPLE BIRTH, PLEASE ANSWER ALL QUESTIONS BY THINKING OF THE BABY WHO WAS BORN FIRST.

2.5 Is your baby male or female?
Male
Female

2.6 Where did you have your baby?[3]
[Drop down box]
Public hospital
Private hospital
At home
Other <please state>

2.7 How many days early or late was your baby?
[Drop down box]
Baby born on due date
Early <days early>
Late <days late>

2.8 How was your baby delivered?
[Drop down box]
Vaginal birth
Elective or planned caesarean
Emergency caesearean

2.9 What was your baby's recorded weight at birth?
<insert weight in grams> or < insert weight in kilograms > or <Pounds and Ounces> >

2.10 What kind of food did your baby receive for his/her first feed after birth?
Breast
Bottle
My baby is not feeding yet

2.11 A) After your baby was born, was your he/she given the opportunity to independently find your breasts by being placed in skin-to-skin contact with you where your baby could move freely? [4]
[Drop down box]
Yes
No (Go to 2.12)
Don't know/can't say (Go to 2.12)

2.11 B) How long after your baby was born was he/she placed in skin-to-skin contact with you?
[Drop down box]
Immediately or within a few minutes
More than a few minutes and up to half an hour
More than half an hour and up to one hour
More than one hour and up to two hours
More than two hours and up to 24 hours
Don't know/ can't say

2.12 How soon after your child was born were you encouraged by a health care professional (doctor, nurse midwife etc.) to breastfeed your child?
Not Encouraged
Immediately or within a few minutes

More than a few minutes and up to half an hour<br>More than half an hour and up to one hour<br>More than one hour and up to two hours<br>More than two hours and up to 24 hours<br>Don’t know/ can’t say
2.13 Has your baby ever had breastmilk? (includes colostrum, expressed breastmilk and breastmilk from a donor or milk bank)<br><br>[Drop down box]<br>Yes<br>No Skip to ***
2.14How soon after he/she was born did your baby first have breastmilk?<br>[Drop down box]<br>Immediately within the first few minutes<br>More than a few minutes and up to half an hour<br>More than half an hour and up to two hours<br>More than two hours and up to 24 hours<br>After 24 hours
2.15While in hospital did your child ever have any other fluids or food?<br>[Drop down box]<br>Yes<br>No<br>Don’t know<br>Did not go to hospital (**skip to section 3**)
2.16 On the day that you left hospital (or at 48 hours if you had a home birth), what most accurately describes how you were feeding your baby?<br><br>Breast<br>Formula<br>Combination of breast and bottle (formula)<br>Expressing breastmilk<br>My baby was not feeding when I went home<br>Can’t remember
**3 Your background with feeding babies**
3.1 How were you fed as a baby?<br>[Drop down box]<br>Just breastmilk and nothing else for at least 4-6 months<br>Mainly breastmilk with some formula<br>Mainly fed with formula with breastmilk only sometimes<br>Just formula<br>Don’t know
3.2 How have your friends and family fed their children when they were babies?<br>[Drop down box]<br>Most of them give formula milk<br>Most of them breastfeed<br>About half of them formula feed and half breastfeed<br>Don’t know

4 Your intentions to feed your baby

The first part of this section is about your intentions to feed your baby BEFORE your baby was born.

4.1 A) Thinking back to before your baby was born, how did you plan to feed him/her?
Breastfeed
Formula feed [skip to 4.2]
Combination of breast and formula feeding
I hadn’t decided [skip to 4.3]

4.1B) Before your baby was born, for how long, if at all, did you plan to give your baby breastmilk?
[Drop down box] Choose most appropriate response
<insert number of> months
Planned to breastfeed for less than 1 month
Planned to breastfeed as long as possible
Planned to have baby determine how long
Didn’t think about how long
Didn’t initially plan to breastfeed

4.2 Why did you think you would feed your baby by this method? (Please write in all reasons)

4.3 How supportive of these plans was your partner/support person?
[Drop down box]
Very supportive
Somewhat supportive
Couldn’t care less
You have no idea of what he/she thinks
Not applicable

4.4 At what stage did you make this decision?

[Drop down box]
Before pregnancy
In the first half of pregnancy
In the second half of pregnancy
After the birth of my baby

4.5 Prior to the birth of your baby, did your partner (or the father of your baby) have any preference for how you fed your baby?

[Drop down box]
Yes, they preferred breastfeeding
Yes, they preferred bottle-feeding
No preference
Don’t know/ Not applicable

4.6 Thinking about the intentions you ticked in Question 4.1 Please indicate your agreement with the following. 7 point scale Anchored by Strongly Disagree and Strongly agree
Breastfeeding is the best way to feed my baby
Breastfeeding is the best option
Bottle feeding is the best way to feed my baby
I wouldn’t change the way I feed my baby
I am committed to this type of feeding

Changing the way I feed my baby would require considerable personal sacrifice
I feel emotionally attached to this type of feeding
This type of feeding has a great deal of personal meaning to me
This type of feeding makes me happy
This type of feeding gives me pleasure

5 Current feeding of your baby
The part asks you about how you are feeding your baby now.

5.1 How do you intend to feed your baby for the next four weeks?
Formula or bottle only
Mostly formula with some breastmilk
Mostly breastmilk with some formula (or other milk)
Exclusive breastfeeding (no other liquids)

5.2 How are you feeding your baby today?
Breastmilk only (no formula or water)
Formula or bottle only
Mostly formula with some breastmilk
Mostly breastmilk with some formula (or other milk)
Expressed breast milk
My baby was not feeding at two days old
My baby is less than 48hours old

5.3 Since this time yesterday, did your baby have any of the following, tick all that apply
[Drop down box]
Breastmilk
Vitamins, mineral supplements, medicine
Plain water
Sweetened or flavoured water
Juice or juice drinks
Tea, herbal tea or infusion
Infant formula
Tinned, powdered or fresh milk (cow's, goat's, other)
Clear broth
Oral rehydration salts
Any solid or semi-solid foods such as mashed veges or fruit, thin porridge, baby cereal, rusks or other foods
Anything else (please specify)

5.4 Thinking about the milk your baby drank yesterday, please mark on the line how much was breastmilk. NOTE THIS IS A SLIDING SCALE from 0 to 100

Only formula or other liquids	Half breastmilk and half formula or other liquids	Only Breastmilk
0		100

6 About your breastfeeding [9]
This section is about your plans to feed your baby

6.1 I plan to breastfeed my child each day for the coming four weeks.
6.2 I intend to breastfeed my child each day for the coming four weeks.
6.3 I will try to breastfeed my child each day for the coming four weeks.
6.4 I am committed to breastfeeding my child each day for the coming four weeks.
6.5 I plan to bottlefeed my child each day for the coming four weeks.
6.6 I intend to bottlefeed my child each day for the coming four weeks.
6.7 I will try to bottlefeed my child each day for the coming four weeks.
6.8 I am committed to bottlefeeding my child each day for the coming four weeks.

7.13 I am satisfied with my breastfeeding experience

7 Support for feeding your baby

8.1 Do you receive any support from friends, family in feeding your baby?
Yes
No GO TO 8.3

8.2 Do you receive any support from health professionals or groups in feeding your baby? [Drop down box]
Yes GO TO 8.3
No GO TO 8.4

8.3 Please indicate the people who have given you support when feeding your baby. Please tick to indicate how much support they have given you. Zero is not applicable.

Baby's father/your partner
Your mother
Other relatives
Your best friend
Other friends
Your doctor
Your midwife/health visitor
Other health professionals (e.g. nurse, nutritionist)
Classes (e.g. childbirth education)
Support groups (IRISH STUDY e.g. Cuidiu (the Irish Childbirth Trust), La Leche League) (Australian Study: e.g. Australian Breastfeeding Association)
Websites/ baby clubs
Mobile phone apps
Written materials/video materials

8.4 Thinking about the most helpful information you received about feeding since your baby was born. Who or what had the most impact on you?

8.5 **Before** your baby was born, did you have enough information about
Breastfeeding YES/NO

Formula feeding YES/NO

Introduction to solids or first foods YES/NO

Other feeding issues
YES/NO

8.6 **After** your baby was born, did you have enough information about

Breastfeeding YES/NO

Formula feeding YES/NO

Introduction to solids or first foods YES/NO

Other feeding issues YES/NO

9 How do you feel about feeding your baby.[6]

Determined by 5.2 for breastfeeders or combination feeders

9.1 Thinking about feeding your baby over the last three months, how do you feel now?

I feel hopeful
I feel hopeful that I have given my baby the best start in life
I feel afraid
I feel anxious that I might not have fed my baby correctly
I feel regret
I feel blameable that because of my actions my baby might not be doing as well as possible
I feel guilty
I feel guilty that I didn't breastfeed as much as I thought I should
I feel challenged
I understood the problems
I feel happy
I feel happy that breastfeeding has worked for me
I am the only person responsible for feeding my child
I feel responsible

Sources

1. Irish Census
2. Irish Household Budget Survey
3. Ireland Perinatal Statistics ESRI
4. Australian Infant Feeding Survey 2010
5. Matich et al
6. Passyn et al
7. De la Mora et al (1999) Iowa Infant Feeding Attitude Scale
8. Vitaliano (1985) Ways of coping checklist
9. Dennis, CL (2003) Breastfeeding Self-efficacy Scale SF
