# Supplementary figures and images for: Understanding breastfeeding behaviours: a cross-sectional analysis of associated factors in Ireland, the United Kingdom and Australia
Source: Int Breastfeed J. 2020 Dec 2;15:103. doi: 10.1186/s13006-020-00344-2 (PMC7709394; doi:10.1186/s13006-020-00344-2)

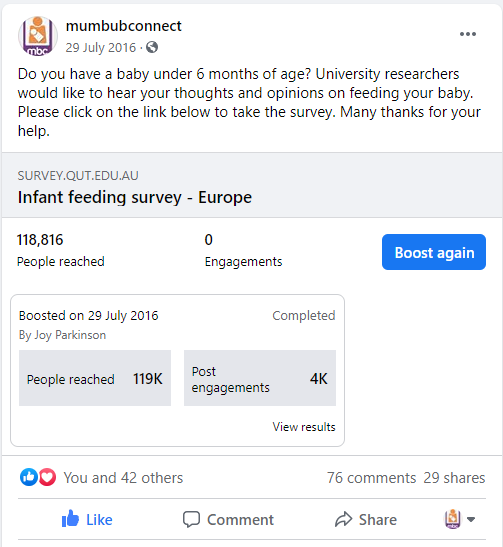

Supplement: Supplementary file 3 — Additional file 3. Example Facebook advertisement. [file 13006_2020_344_MOESM3_ESM.docx]
